# From Movement to Market: A Policy Analysis of Swedish Domestic Violence Shelters (1980–2024)

**DOI:** 10.1177/10778012261440212

**Published:** 2026-04-13

**Authors:** Felicia Forthmeiier, Veronica Ekström, Johan Vamstad

**Affiliations:** 1 Department of Social Work, 531597 Marie Cederschiöld University, Stockholm, Sweden; 2 Center for Civil Society Research, Marie Cederschiöld University, Stockholm, Sweden

**Keywords:** Women’s Shelter Movement, domestic violence shelter, policy analysis, gendered violence, governmentality

## Abstract

Domestic violence shelters are a vital service for abused women and children. The Women's Shelter Movement has historically provided this support. Recently, Sweden has shifted toward marketizing shelters, increasing demands for formalization, and allowing for-profit organizations. This study analyzes policy documents from the 1980s to the present to examine the causes of this shift, its implications for the Women's Shelter Movement, and the accessibility of shelters for abused women. The findings show that the role of women's shelters has changed; public policy has redefined the movement, and reforms have made shelters less accessible for abused women.

## Introduction

The Women's Shelter Movement started as a low-threshold organization, ideologically driven by feminist ideas of sisterhood, to bring about change for both abused women individually and for a society free of violence (Hague, 2021). Since then, shelters have become institutionalized and professionalized (Brückner, 2001; Dewey & Germain, 2014; Wies, 2008).

Sweden has a population of 10.5 million. In 2023, nearly 11,000 Swedish women and children spent at least one night in a shelter (National Board of Health and Welfare [NBHW], 2024). Initially, women with children could go directly to shelters for protection. Today, shelters are provided by local social services and regulated by the Social Services Act (SSA; SFS 2025:400). Municipalities can procure services from private companies, a common practice in social services. Most services, including shelters, require a formal needs assessment. However, there are still a few nonprofit women's shelters where women (albeit without children) can turn directly to find sanctuary from violence (Roks, 2025). Both public and private organizations provide services in competition; however, almost all services are fully paid for by municipal social services (Ekström & Hvenmark, 2025).

The marketization of shelters has surged, with the share of private for-profit shelters rising from 8% in 2012 to 47% in 2023, while the share of shelters run by the Women's Shelter Movement declined from 71% to 46%. Public shelters are a small portion (NBHW, 2013, 2024). Shelter in Sweden is thus a formalized, commodified service in a welfare market (Ekström & Hvenmark, 2025). The largely unregulated market has significant variation in form, size, and structure, indicating inconsistent shelter quality and unsatisfactory rights for children (Ekström & Hvenmark, 2025; Lauri & Lauri, 2024). However, in 2024, a governmental reform of shelters was implemented in response to quality issues and concerns regarding children's rights. Today, shelters are required to meet established quality standards and obtain a license to operate. The new shelter reform promotes professionalized and institutionalized shelters embedded in the social services system. Andersson et al. (2024) show that, from the perspective of abused women, the institutional conditions surrounding shelters appear complex and challenging to navigate, which might hinder women's access to protection.

The aim of this study is to explore how the protection of abused women went from being provided by an inclusive and ideologically driven social movement to becoming a formalized service in a welfare market. To accomplish this, we analyze how the Women's Shelter Movement and abused women's need for protection have been articulated in official government documents, and how this articulation has influenced how and by whom shelters are provided for abused women and their children. The study is thus a case of the professionalization and marketization of domestic violence shelters in Sweden. It provides insights into how policy frameworks, not only in Sweden, can shape advocacy for women's rights in general, and domestic violence shelter services in particular.

## Previous Research

### Shelters—From a Feminist Movement to Professional Social Services

The first shelters in the United Kingdom and United States opened in the early 1970s, rooted in the feminist movement (Elman, 2003). They aimed to provide abused women with a refuge from violent men, where they could plan and establish new lives. The shelter movement combined practical support with political activism to raise men's violence against women from a private to a political issue (Hague, 2021). Research has shown how perceptions of men's violence against women constitute an important aspect of how shelter work is organized (Brunell, 2002; Diner & Toktaş, 2013). Shelter services are in some countries understood as related to other sources of support, such as transitional housing, part of the housing market, or a part of the welfare system (Baker et al., 2009). International agreements such as the Convention on the Elimination of All Forms of Discrimination against Women (United Nations General Assembly, 1979), the UN Declaration on the Elimination of Violence against Women (United Nations General Assembly, 1993), and the Council of Europe's Istanbul Convention (2011, in force in Sweden in 2014) have also guided policy. The Istanbul Convention is the first binding European treaty on violence against women and the broadest global intervention framework (Stubberud et al., 2018).

Research has shown how demands tied to funding transform shelters into more service-oriented, individualized practices (Fleck-Henderson, 2017; McDonald, 2005; Warrington, 2003; Wathen et al., 2015). Advocacy against domestic violence has become less radical as organizations professionalize (Wies, 2008). Shelters now rely more on paid staff due to funding requirements, thus reducing the role of volunteers (Brückner, 2001; Dewey & Germain, 2014; Warrington, 2003). As a result, shelter activists with diverse backgrounds are being replaced by trained employees, as shelters trend toward institutionalization, professionalization, and de-radicalization (see also Elman, 2003).

These international developments are also reflected in Swedish shelter practices. Eduards (2002) noted that in the 1990s, public shelters in Sweden prioritized professionalism and expertise, while nonprofit women's shelters emphasized solidarity and empathy. The study by Helmersson (2017) found that support and interventions for abused women and children were being renegotiated due to the ongoing professionalization of the Women's Shelters Movement during the 2010s. This shift was driven by legal requirements and policies, making the movement less independent and more of a professional service provider. Swedish shelters, like those in many countries, have become institutionalized, professionalized, and marketized (Ekström & Hvenmark, 2025; Lauri & Lauri, 2024).

Ekström and Hvenmark (2025) note that Swedish non-profit, feminist-based shelters face challenges in a market-driven environment. Securing public funds can bring stability and improve efficiency and innovation, but it risks compromising core values. Shelters that successfully secure public funding may achieve greater financial stability, which can enhance efficiency and innovation in service delivery. However, this competitive landscape poses risks to their original missions and values. For example, shelters prioritizing full-time staff over volunteers might advocate more effectively for victims of IPV but may also create internal tensions by straying from their core values. Conversely, shelters that rely heavily on volunteers may feel pressured to soften their critical stance on funding to maintain financial support. Funding can lead to a focus on simpler cases, neglecting complex needs, and result in shorter, standardized placements instead of tailored support that shelters typically view as essential (Ekström & Hvenmark, 2025).

### Welfare Services in Transformation—New Public Management

The New Public Management (NPM) is a widespread governance paradigm within Swedish public administration, which promotes liberal ideas of marketized welfare systems (Hasselbladh et al., 2008; Lundström & Wijkström, 2012; Sallnäs & Wiklund, 2018). This governance model promotes notions of efficiency, measurability, documentation, and standardization, emphasizing service quality (Agevall et al., 2017). Competition in welfare markets can also lead to quality deficiencies, for example, by leading to profit getting priority over actual quality (Hartman, 2011).

Marketization involves procurement processes in which service providers are selected based on both price and quality (Stenius & Storbjork, 2020). While price just reflects costs, quality is complex and subjective, shaped by perceptions and conditions, making it difficult to define and measure (Harvey & Green, 1993; Hjärpe, 2017). Understanding prevailing notions of quality is vital in the context of Swedish shelters, as these perceptions can influence not only the design of procurement processes and the evaluation of tenders but also the assessment of individual shelters and their operations. Widely accepted views of quality can even shape broader concepts of what constitutes a shelter, how it should function, the conditions it operates under, and ultimately, its long-term viability.

Quality in social work lacks a single definition (Blom & Morén, 2012). Although no uniform definition of shelter quality exists, there have been attempts by the government to identify shelter quality characteristics, for example, by assigning the National Board of Health and Welfare (NBHW) the task of developing both indicators and regulations to guide, control, and monitor the quality of shelter (Ds 2012:52; HSLF-FS 2025:3). Quality can relate to power, as those defining it prioritize specific quality characteristics (Gross et al., 2015; Megivern et al., 2007; see also Hjärpe, 2017). Research also shows that social work involves crucial quality aspects that are hard to measure (Evaldsson, 2024; Lorenz et al., (2020)). Lorenz et al. (2020) note conflicts between NPM's rigidity and social workers’ need for flexibility, while Evaldsson (2024) emphasizes interpersonal encounters and client needs as key quality characteristics.

Requirements for licensing aim to ensure high-quality shelters, but research by Sallnäs and Wiklund (2018) shows that licensing of Homes for Care and Housing (HVB) does not necessarily lead to better treatment outcomes or market regulation. A study by Pålsson et al. (2022) found that licensing mainly affects standards, rather than client experiences or staff skills. According to Davidson (2009), licensing can be seen as government gatekeeping, while Power (1999) suggests that it shapes applicants and the market. Pålsson (2018) notes that licensing prioritizes measurable indicators over personal values. Although intended to eliminate unsuitable providers, licensing does not prevent all inappropriate ones. A study by Lundström and Shanks (2022) found that HVB standards are relatively low, but some management qualifications have improved over time.

## Theoretical Approach

Carol Bacchi (2009) argues that government documents and proposals define the problems that policymakers focus on, making them instruments for governing, not just neutral descriptions of the policy process. The “problems” are not objective; they do not merely exist awaiting resolution; instead, they can be understood as being constructed to create meaning and impact for policy proposals. Thus, by presenting solutions and suggesting that something needs to change, policies can be seen as shaping problems rather than solving them (Bacchi & Goodwin, 2016).

Within the What's the Problem Represented to be (WPR) framework, problematization is central to *governing* and policymaking, serving as a tool for government practice (Bacchi, 2009). This theoretical approach to Governing is known as *Governmentality*, and it enables an understanding of how power is executed through different forms of governance, not only through direct control and coercion (Foucault, 1991). Instead, power can be projected by influencing the population's behaviors, values, norms, and identities, as well as civil society organizations and other societal actors through discourses and policy governance (Dean, 2010). Hence, according to the theory of governmentality, the government can “shape in some way who and what individuals and collectives are and should be” (Dean, 2010, p. 20).

In this study, problem representations in policy documents are understood as arguments for measures like formal requirements and legal regulations. By uncovering how the government describes challenges and problems linked to support and protection for abused women and children, we hope to better understand how this has subsequently influenced the development and formalization of women's shelters.

Bacchi and Goodwin (2016) suggest the following questions when drawing a WPR analysis: (1) What's the “problem” represented to be in a specific policy? (2) What assumptions underlie this representation of the “problem”? (3) How has this representation of the “problem” come about? (4) What is left unproblematic, and where are the silences? (5) What effects are produced by this representation of the “problem”? and (6) How and where has this problem representation been produced and defended? Bacchi and Goodwin (2016) describe the questions as a malleable framework. They should not be viewed as a strict formula to be followed, but rather as a guideline for critically engaging in matters of policy and governance. This analysis focuses on the first five questions of the framework: problem representations, assumptions, the solutions presented, their impact, and what is left unsaid.

## Methods

### Sample

This policy analysis comprises 20 official government documents that address shelters to various extents. The study is limited to documents issued by the government or on its behalf (SOU, Ds, Prop, Skr., and Draft Legislative Council Referral) from 1980 to 2024. Swedish Government Official Reports (SOU, Statens offentliga utredningar) are reports produced by government-appointed committees or investigators, often forming the basis for future policy proposals. Ministry Publication Series (Ds, Departementsserien) are reports generated by individual ministries (departments) that represent internal governmental activities and often support forthcoming reforms. Government bills (Prop, Proposition) constitute the official legislative proposals submitted to the Parliament and serve as explicit expressions of political intent. Government Communication (Skr. Skrivelse) functions to inform the Parliament about the government's actions and intentions and reflects its strategic direction. Lastly, Draft Legislative Council Referral (utkast till lagrådsremiss) is the government's proposal for new reforms and laws, sent to the Council on Legislation for review before being presented to the Parliament. Thus, the sample includes several types of policy documents; however, the common denominator is that they all represent the government's policy orientation and intentions, thereby influencing policy development both directly and indirectly.

The first mention of women's shelters (or Women's houses, as they were called) dates to 1980, marking our starting point. The endpoint of the study is 2024, the year a new governmental reform regulating shelters came into force. The reform introduced licensing requirements and formal quality standards, marking a significant shift in who holds the authority to define what constitutes a shelter and what such services should include.

### Procedure

A purposive sampling method was employed to collect the data, using the keywords “Women's Shelter Movement” [kvinnojour], “shelters” [skyddat boende], and/or “social service provider” [utförare] as inclusion criteria. The policy documents included first were those that are widely known in the Swedish policy area on violence, for example, the *Women's Peace Bill* (Prop 1997/1998:55) and *A Window of Opportunity: Strengthened Rights for Children in Shelter* (SOU 2017:112). A snowball approach was then used, with the empirical material guiding the identification of additional policy documents (Bryman et al., 2025).

### Analytical Method

The study's theoretical approach and analytical framework are closely linked. Bacchi's “What's the Problem Represented to be” (WPR) is a theoretical approach to understanding governance, governmentality, and policy as tools for achieving goals, exercising control, and exerting influence. It is also, however, an analytical method for exploring policy.

Text relevant to the research question was distilled from the policy documents and collected as raw empirical data. The data was then closely read to gain an initial understanding of recurring themes and visible patterns. The Nvivo software was used for coding. The coding was based on five of Bacchi's questions. In this way, the material was naturally sorted into categories of problem representations, solutions, assumptions, silences, and consequences. The categorization was then sorted in chronological order to distinguish when specific problem representations appeared, which formed the basis for the four time periods presented in the results section (see Table 1). All direct quotations have been translated by the authors.

**Table 1.** Summary of Results Based on WPR Analysis.

	The personal is political 1980–1993	The nonprofit expertise—confirmed and challenged 1994–2003	The establishment of the shelter market 2004–2013	Policy redefining the Women's Shelter Movement 2014–2024
What's the “Problem” Represented to be?	Domestic violence is a societal issue; thus, the welfare state must support abused women	The voluntary efforts of women's shelters are being questioned regarding their sufficiency for victims of violence	The delineation of responsibilities between women's shelters and the public sector remains ambiguous	Regulating the management of shelters and the necessity for enhanced quality standards
Assumptions	Women's shelters cannot help the most vulnerable women	Women's shelters are essential for public welfare	Market solutions organize welfare effectively	Licensing requirements lead to improved shelter quality
Solutions	Assigning service provision for abused women to the public welfare	Enhanced financial support for women's shelters	Conditional grants steered toward the institutionalization of shelters	Implementing shelter law and licensing requirements
Silences	Whether the nonprofit form adds value in itself	Whether the nonprofit form adds value in itself	Whether the movement wants to become a professional welfare service provider	The impact of shelter legislation on women's shelters’ ideology
Consequences for women's shelter	The women's shelter has interpretive precedence on what abused women need and how to organize shelters	The movement is still the primary provider of shelters, but it relies on government funding	The movement oscillates between the roles of expert and provider	License requirements and quality standards for shelters challenge the movement's ideological impetus

Trustworthiness was sought through thick descriptions of the policy texts, where results and analysis aimed at transparency and visibility. This enables the reader to follow the analytical chain (Lincoln & Guba, 1985; Nowell et al., 2017).

## Results

The result is presented chronologically, divided into four periods: (1) The Personal is Political, 1980–1993, (2) The Nonprofit Expertise—Confirmed and Challenged, 1994–2003, (3) The Establishment of the Shelter Market, 2004–2013, and (4) Policy Redefining the Women's Shelter Movement, 2014–2024. Table 1 provides a summary of the results.

### The Personal Is Political (1980–1993)

So-called “Women's houses” were first mentioned in a Government Official Report (SOU) from 1980 (SOU 1980:44), which marked the starting point of the political interest for the nonprofit Women's Shelter Movement in Sweden. These organizations supported activities for women's personal and collective liberation. The report suggested that women's houses should be publicly funded due to their important work: “by placing women's problems at the center and driving forward the work for greater equality between women and men, several of the women's organizations make such significant contributions to society that they should not be excluded from the regular state support for social movements” (SOU 1980:44, p. 66).

In the Ministry Publication Series (Ds S 1983:2) from 1983, domestic violence was described as a societal problem (Ds S 1983:2), and the Women's Shelter Movement was acknowledged for playing a vital role in exposing men's violence against women and moving domestic violence from a domestic to a societal setting. This underscored the essential role of women's shelters in society.

As domestic violence shifted from being a private matter to a societal issue, abused women became the responsibility of public authorities, specifically local social services. Medical care, housing, and psychological support were identified as the needs of abused women (Ds S 1983:2). At that time, the term “shelters” was not used. However, it was acknowledged that women and children fleeing domestic violence required safe housing: “a battered woman who has been forced to flee her home, perhaps with her children, needs accommodation where she can feel safe and where she is not left alone with her fears and worries” (Ds S 1983:2, p. 135).

The Women's Shelter Movement was vital for some abused women, but not all. Vulnerable, resource-poor individuals were primarily the responsibility of local social services. The report stated that nonprofit women's shelters alone could not meet all local emergency needs for women facing violence. As social services took on responsibility for victims of violence, they needed to create an organization tailored to serve the target group, which, according to the report (SOU 1993:82), women's shelters could not adequately cater to. Nonetheless, nonprofit shelters remained the primary provider of shelter services, and their importance grew during the 1980s (SOU 1993:82).

In the Government Official Reports 1993:82, the voluntary sector was portrayed as an essential complement to public sector services. A study of voluntary organizations showed how social movements were a meeting place for forming opinions and voicing demands related to social issues. The Women's Shelter Movement was even described as, in some cases, superior to the public alternative (SOU 1993:82): “In some cases, the voluntary option has also proven to be superior to a governmental option. We are thinking, for example, of women's shelters” (SOU 1994:139, p. 390).

In summary, the early work of the nonprofit Women's Shelter Movement significantly impacted society's understanding of men's violence against women. The movement successfully shifted the perception of domestic violence from a private issue to a political one, and its advocacy aimed to ensure that resources were allocated to protect and support victims of violence. Their arguments appeared to resonate with policymakers, as policy documents acknowledged that social services bear ultimate responsibility for abused women and children. The government's admission of responsibility did not diminish the importance of women's shelters. On the contrary, women's shelters were regarded as an essential complement to public welfare during the latter part of this period.

### The Nonprofit Expertise—Confirmed and Challenged (1994–2003)

The number of nonprofit women's shelters in Sweden increased significantly from the late 1970s to the 1990s. In the 1990s, a shelter was defined as a place for abused women and their children, both for emergencies and for planned placements. Most women seeking help had experienced physical and emotional abuse or threats of violence. Shelters became a crucial intervention to prevent future violence (SOU 1994:139).

Many nonprofit women's shelters were known for their small facilities and limited space. These shelters typically did not turn away abused women and their children in need, which sometimes resulted in overcrowding. Consequently, shelter staff and volunteers occasionally offered a room in their own homes as a temporary solution (SOU 1995:60). The limited availability of shelter services was highlighted, with special mention of the risk that not receiving personal contact could make the woman change her mind and return to the abuser (SOU 1994:139): “Instead, she needs a personalized contact, and ideally, the contact should be established immediately. There is an imminent risk that the woman will otherwise change her mind and choose to remain with the man who abuses her” (SOU 1994:139, p. 175).

The documents indicated that the Women's Shelter Movement's extensive work with abused women made them experts in the field (Prop 2000/01:79; SOU 1995:60). Ninety percent of the local social services turned first to the Women's Shelter Movement when they needed to organize shelter for women exposed to violence (SOU 1995:60).

The work at shelters was depicted as demanding, raising concerns regarding insufficient staffing, many of whom had only part-time jobs. Despite this, the nonprofit women's shelters were generally considered to have a high level of accessibility, thanks to the commitment of their staff and volunteers. While this was seen as positive, there were also concerns about the risk of occupational burnout (SOU 1995:60):

Many [nonprofit] shelters would, therefore, benefit from having a staff member employed, at least part-time. Additionally, given the psychological strain of on-call work, it is beneficial for the center to have more than one employee. The opportunity to share experiences reduces the risk of exhaustion and the consequent attrition of on-call workers. (SOU 1995:60, p. 175)

The promotion of professionalized shelters appeared to align with a much broader political effort to privatize the provision of publicly funded welfare services in the 1990s (SOU 1993:82; SOU 1994:139). The professionalization and formalization of shelters made them better suited for the type of welfare market that was beginning to emerge. An important starting point in the renewal of the public sector was to allow for-profit and nonprofit actors to take greater responsibility for delivering services (SOU 1994:139).

The increased trend toward private care providers justified a review of the rules governing the licensing and supervision of private providers. It was proposed that the same quality requirements should apply regardless of whether the activities were public or private. In addition, it was suggested that licensing requirements would be introduced for “professional private full-time activities providing care, treatment, nursing or supervision for children, adolescents and adults” (SOU 1994:139, p. 539). It was noted that women's shelters also provided full-time residence to complement the local social services and that they “are often run with voluntary efforts but sometimes also with professionals in charge of the activities” (SOU 1994:139, p. 545). However, according to the Government Official Report, these organizations would not be covered by the legal requirements for private providers (SOU 1994:139), as women's shelters should retain their nonprofit character and operate independently (Prop 1997/98:55).

The shift to marketized welfare required a clearer division of roles and responsibilities (SOU 1994:139). To define local social services’ roles for victims of violence, amendments to the SSA were suggested (Prop 1997/98:55). This proposal aimed to address flaws in local services, where domestic violence was often deprioritized, leading to insufficient support for abused women. This change in responsibility also impacted the Women's Shelter Movement's volunteer services. The 1997 bill not only strengthened the mandate of local social services but also indicated that social work in this area was overly dependent on nonprofits and volunteers (Prop 1997/98:55).

Despite these clarifications, the political ambition to care for abused women remained ambiguous. The SSA clearly stated that social services were primarily responsible for providing shelter and support to abused women. The local social services were, however, almost entirely dependent on the Women's Shelter Movement to provide shelters and support for abused women. The mere existence of the Women's Shelter Movement thereby became indicative of the government's failure to take responsibility for the needs of victims of violence (SOU 1995:60).

Public funding for the nonprofit women's shelters can also be understood as contributing to a lack of clear division of responsibilities between the nonprofit and the public. The funding aimed to ensure that the women's shelters could offer adequate support, hire staff, and enable general organizational development. Thus, the availability of shelters increased, and more women could be helped (SOU 1995:60). The government bill (Prop 1997/98:55) proposing increased funding to women's shelters also stated it was crucial to safeguard the movement's freedom and independence: “It is important that the organizations [women's shelter] can retain their voluntary characteristic and exist on their terms” (Prop. 1997/98:55, p. 61). Local and governmental authorities were informed to exercise caution when setting unilateral conditions as grant requirements (Prop 1997/98:55).

Although official government documents about the Women's Shelter Movement mainly focused on abused women, they also acknowledged the needs of children exposed to violence in the family. Many women had accompanying children at shelters. Shelters, facing limited resources, sometimes lacked the resources to help these children. A 1994 survey found that 1,500 women and 1,300 children spent at least one night in a shelter the previous year. However, the needs of children were not addressed in the 1990s government bill, which only stated a need to review their conditions later (Prop 1997/98:55).

In summary, the Women's Shelters Movement had the authority to interpret the needs of abused women based on their expertise and role, alongside local social services. However, challenges arose regarding the reliance on volunteer work, prompting questions about its adequacy. This led to increased public funding for staff and better accessibility, reflecting progress toward professionalization. These changes occurred amid a broader shift to a more market-driven welfare system, which was still unrealized in shelter services at the time.

### The Establishment of the Shelter Market (2004–2013)

Given its expertise, the Women's Shelter Movement was deemed the authority on defining violence against women and organizing protection for abuse victims (SOU 2004:121). It was the primary provider of shelter services, with some voluntary and municipal alternatives (Prop 2006/07:38). However, in the Government Official Report 2004:121, a shift was noted, and the report highlighted a trend toward institutionalization, viewed as a positive development necessary for future progress (SOU 2004:121, p. 16). Institutionalization meant integrating victim support within established authority structures, routines, and resources (SOU 2004:121).

The need for differentiated shelters for vulnerable groups was again represented as a problem, for example, for women with substance use disorders or disabilities, which was highlighted in the government bill 2006/07:38. While various shelter options existed in the local market, choosing among them posed challenges. Economic incentives were portrayed as a problem that resulted in the selection of “lighter” services instead of the more expensive shelters that could better address women's needs (Prop 2006/07:38; SOU 2006:65).

Another consequence of the increased options for social services to externally place abused women was that some placements were highly questionable; they involved unsuitable environments, such as hostels or hotels, rather than women's shelters. The Government Official Report (SOU 2006:65) did not clarify if economic incentives or knowledge gaps contributed to this, but it highlighted security issues, children's rights, and quality assurance concerns. This suggests a disconnect between the needs of abused women and the social services’ perception of protection for victims of violence. The government bill (Prop 2006/07:38) noted that the definition of shelter used by social services often differs from that of women's shelters. “…social services [have] used hostels or similar setups, which in many cases must be considered less appropriate in this context. It is, of course, questionable whether this constitutes a ‘shelter,’ depending on how it is defined” (SOU 2006:65, p. 132).

As women's shelters professionalized, relying less on volunteers and hiring more staff, questions arose about monitoring, evaluation, and quality assurance (SOU 2006:65; SOU 2007:66). Women's shelter representatives felt the municipality lacked follow-up and knowledge of the support services. Renewed discussions on statutory licensing and supervision commenced. However, a Government Official Report (SOU 2006:65) noted diverging opinions, arguing that women's shelters are too unique to meet conventional long-term care and treatment standards required for licensing private providers. “It should be emphasized that [women's] shelters can be very different, ranging from well-established and developed services with paid staff to shelters run entirely by volunteers. It is doubtful that the latter would fulfill the requirements of professionalism, long-term care, and treatment. However, they may be just as important” (SOU 2006:65, p. 152).

According to the SSA, publicly funded social services must be of good quality. This includes women's shelters in cases where the local social services place abused women and children in shelters. The Government Official Report (SOU 2006:65) argued that such quality requirements for nonprofit women's shelters should be seen as a measure to improve the status of their work: “To the extent that social services use the services of women's shelters to provide support for abused women and their children, the quality requirement should also cover this. It is also about raising the status of women's shelters and giving them the recognition they deserve for their tireless support to women and children exposed to violence” (SOU 2006:65, p. 152).

The division of responsibility for abused women and their accompanying children between local social services and the Women's Shelter Movement remained unclear. A Government Official Report (SOU 2006:65) described tensions regarding uncertainties and a lack of clarity about responsibilities, expectations, and services (SOU 2006:65). In the government bill (Prop 2006/07:38), it was proposed that a mandatory requirement for the social services to take responsibility for victims of violence should be included in the SSA. Still, the role of nonprofit women's shelters was not to be dismissed, as they contributed essential added value that was worth preserving (Prop 2009/10:55):

In this context, it is important to point out that the work carried out by voluntary organizations is an important complement to municipal social services and is usually based on solid knowledge and experience in the field. Women can turn directly to a voluntary organization. For various reasons, some women do not want to turn to social services, and for them, the work of voluntary organizations is crucial. (Prop 2006/07:38, p. 12)

This ambiguity also pertains to the funding of shelters, which adds to the confusion regarding who is accountable for protecting abused women and children. Policy documents of this period stated that the women's shelter should be funded by government grants (Prop 2005/06:155; Prop 2006/07:38; SOU 2004:121). The purpose of the public funding was to continue to ensure the role of the shelters of the Women's Shelter Movement as a complement to social services. It was also argued that efforts to combat men's violence against women required a strong Women's Shelter Movement (SOU 2004:121; SOU 2007:66). Still, how the parties were to understand their different responsibilities was made unclear by the fact that policy, rhetoric, and practice differed: “For example, there is a demand for municipalities to take greater responsibility and step up their efforts to help women who are victims of violence. At the same time, there is a demand for more resources for the Women's Shelter Movement. Who should complement whom, and who is actually responsible?” (SOU 2006:65, p. 149).

Public funding for the Women's Shelter Movement was considered crucial for protecting abused women. However, it was asked rhetorically what other services would be funded without quality assessment (SOU 2006:65). Local social services raised concerns about the value of their public funding and their lack of insight into the nature of nonprofit women's shelters, indicating a trend toward control over how these funds were spent. A conventional view of public funding emerged, emphasizing the municipality's focus on value for money (SOU 2006:65; SOU 2007:66). Another example of the government's growing control over shelter services was when, in 2011, the Swedish National Board of Health and Welfare received a government assignment to define shelter services, assess the sector's size and quality, and propose a quality development model (Ds 2012:52).

In summary, shelter services faced growing scrutiny as roles became clearer: local social services had the ultimate responsibility, while the Women's Shelter Movement shelters became contracted providers. This division was akin to that of other marketized welfare services, raising concerns about quality, cost-effectiveness, control, and oversight.

### Policies Redefining the Women's Shelter Movement 2014–2024

In a Government Official Report from 2014 (SOU 2014:49), it was suggested that the municipalities’ responsibility to provide shelter services should be regulated in the SSA and that all shelter providers should be required to have a government-issued licensing permit (a proposal that was not implemented until 2024). The same Government Official Report (SOU 2014:49) proposed that the specific needs of all women and accompanying children should be at the center of shelter placements.

Even though the Women's Shelter Movement was recognized for providing high-quality services and for its knowledgeable and engaged volunteers, the problem representations in the policy texts still concerned the unclear division of responsibility between the local social services and the Women's Shelter Movement (SOU 2014: 49):

Shelters are in the borderland between the private and the public, between nonprofit organizations and public policy, ideology, and municipal finances. […] However, the direction is clear: the path is moving from volunteering to professionalization, from volunteerism to increased regulation and public responsibility in shelters. (SOU 2014:49, p. 84)

The Government Communication of the national strategy against men's violence toward women (Skr. 2016/17:10) clarified that women's shelters collaborating with local social services must follow their regulations, including quality assurance, confidentiality, and case documentation (Skr. 2016/17:10). This defined the Women's Shelter Movement's formal transition from a movement to a professional welfare service provider (SOU 2014:49).

Discussions on shelter licensing requirements evolved. Proposals were made regarding legal regulation and the establishment of licensing. The main argument was that children's rights must be ensured while living in shelter, as children had been treated as appendages to their caregiver rather than as legal subjects in their own right. Overall shelter quality was also discussed. Reform arguments pointed out the lack of licensing, which enabled unscrupulous actors to operate shelters. The sector's diversity hindered a national overview of shelter availability (SOU 2017:112).

In official government documents from 2014 and 2019 (Ds 2019:7; SOU 2014:49), it was argued that women's shelters could decide whether or not to continue providing shelter as a professional service (with licensing) on behalf of local social services. They had the option, it was argued, not to do so, as each women's shelter operated as an independent organization (Ds 2019:7; SOU 2014:49). It was also argued that the Women's Shelter Movement would likely develop shelter services as a “branch” of the association's offerings, probably leading to increased specialization and improved service quality. This professionalization could thus be compartmentalized within the shelter branch, allowing other services, such as advocacy and non-residential counseling, to remain unaffected. This was already occurring in more advanced women's shelters (SOU 2014:49). However, a continuing challenge to the advancement toward regulation and professionalization was that the Women's Shelter Movement appeared to lack interest in operating shelters commercially (SOU 2017:112).

In the Government Official Report (SOU 2017:112), an impact assessment concluded that most Women's Shelter Movement shelters had good prospects of continuing to operate but might require improvements in service quality to meet the new standards. The assessment overlooked other nonprofit qualities, such as the special added value and ideology of the Women's Shelter Movement (SOU 2017:112). Representatives from the Women's Shelter Movement highlighted their unique role in complementing local social services. They raised concerns about proposals for shelter legislation, increased supervision, and licensing permits (SOU 2014:49). The proposal left room for nonprofit women's shelters to continue offering shelter for unaccompanied women seeking support outside the system. However, it remains unclear why this target group was not included in the new quality requirements or how the women's shelters would be able to fund their shelters without overnight compensation from the municipality (Ds 2019:7; SOU 2017:112).

It is worth noting that in the preparatory work for the new shelter reform (Prop 2023/24:31; SOU 2017:112), the semantics also shifted, where s*helter*, which was previously synonymous with women's shelters and the Women's Shelter Movement, was now described as a separate intervention, independent of the type of provider.

With the establishment of the shelter market, shelter was defined as an economic activity that no longer qualified for public grant funding. This forced local governments to distinguish between procured shelter services and grant-funded open activities, such as advocacy, counseling, and violence prevention. This separation proved challenging for the Women's Shelter Movement organizations, which were closely integrated. Moreover, many shelters were small, typically providing only one to three overnight accommodations. “This means that, in many cases, staff employed in the ‘non-residential part of the organization’ are also engaged in the shelter, since they often work across counseling, advocacy, and shelter services” (Ds 2019:7, pp. 80–81).

Hence, the official government documents legitimize the proposal for shelter reform by arguing against unscrupulous actors, emphasizing the need for extensive quality assurance, and highlighting children's rights. They did not discuss the risks associated with regulating the market, for example, compromising the independence, experience, and the nonprofit nature of the women's shelters, which were earlier considered the movement's strengths, while forcing them into a formal system that offered no room for them.

Quality in shelter services was proposed to encompass adequate staffing and safety devices, accommodations for children and their needs, and personnel with the appropriate training and expertise (SOU 2017:112). Measurable quality indicators, statutory requirements, and government funding for implementing systematic quality management systems further regulated quality efforts (Draft Legislative Council Referral, S2022/03649; Ds 2019:7).

The proposals about regulation, quality requirements, licensing, and professionalization of shelters had been underway for several decades when they finally, in 2023, led to a government bill titled, S*trengthening the rights of children and adults in shelter* (Prop 2023/24:31). The government argued this was a signal of growing ambitions for government policies in the area (Prop 2023/24:31). The preparatory work (Draft Legislative Council Referral, S2022/03649) for the bill (Prop 2023/24:31) stated that there were no regulations on what shelter services could include or how unethical operators could be prevented from providing shelter services: “there are thus no regulations on what shelters should include as a minimum, nor are there requirements for quality and safety in the activities concerned, which makes it difficult for municipalities to ensure good quality service for the individual” (Draft Legislative Council Referral, S2022/03649, p. 73).

Shelters were now defined as secure housing with targeted interventions for safety, including locks, alarms, support calls, counseling, safety planning, and practical assistance (Prop 2023/24:31). This definition mandated professional staff. While the government bill (Prop 2023/24:31) allowed shelters to engage volunteers, these were not allowed to replace professional staff.

On February 16, 2024, the Parliament voted in favor of the government bill (Prop 2023/24:31). This decision meant that shelter services were legally defined and regulated in the SSA. Moreover, accompanying children would henceforth be placed in shelters based on their own placement decisions, not only as passive appendages to their mothers. A government-issued license linked to quality standards was also introduced as a requirement for operating a shelter for victims of domestic violence.

## Discussion

In the 1990s policy documents, the Women's Shelter Movement was recognized as experts on men's violence against women, and key providers of shelter (Prop 2000/01:79; SOU 1995:60) However, their volunteer work faced scrutiny, highlighting heavy shelter work and the risk of occupational burnout among staff (SOU 1995:60). A proposed solution was to hire more employees to allow staff to share experiences and reduce the risk of exhaustion. Though evidence for this proposal was not discussed, the proposal aimed to transition from a volunteer to a professional organization.

The problem representation of the “burnout risk” in women's shelters suggested the need to hire additional staff to support the effort of institutionalizing shelters, as noted in the Government Official Report, SOU 2004:121. This reflected the NPM paradigm in Swedish welfare, which emphasized cost-efficiency and performance management (Hasselbladh et al., 2008; Hwang & Powell, 2009; King, 2017). However, to apply NPM principles, shelters must ensure at least a minimum standard of professionalism and not rely too heavily on volunteers. Second, we can understand the shift toward institutionalization as aiming to integrate shelters into the public welfare system. While the Women's Shelter Movement performed voice and advocacy for abused women (SOU 1993:82), it did not relieve the social services from their obligation to support and protect them. With NPM policies promoting market solutions and private providers in public welfare, the institutionalization of shelters could be interpreted as a way to align shelters to the social services’ rules and requirements. This was a requirement for municipalities to procure women's shelters when they had not managed to establish publicly operated shelters.

The policy reports framed institutionalization as vital for supporting abused women but neglected the consequences of limiting nonprofits’ ability to shape interventions based on feminist principles (see also Fleck-Henderson, 2017; McDonald, 2005). Notably, in 1994, it was stated that support from a nonprofit organization could be superior to government-administered support (SOU 1994:139), in 2000, women's shelters were recognized as leading experts in the field (Prop 2000/01:79), in 2004, it was claimed the shelter movement defined the needs of abused women and shaped their interventions accordingly (SOU 2004:121), and in 2009, mission-driven organizations were acknowledged for their significant added value (Prop 2009/10:55).

A subsequent problem representation states that women's shelters did not meet the needs of vulnerable groups, like those with substance use disorders or disabilities. Additionally, some victims were so traumatized that they needed around-the-clock support (Prop 2006/07:38; SOU 2006:65). This raised concerns about the effectiveness of women's shelters despite previous accolades. It suggests a political shift toward the professionalization of shelters, where they were becoming formalized services designed around social service needs, diverging from the ideals of the Women's Shelter Movement.

This was confirmed not least by the alarming reports about social services saying that, during the mid-2000s, they sometimes placed abused women and children in hostels, campgrounds, and other less suitable housing facilities (SOU 2006:65). It indicates that there was a significant knowledge gap at the time between the social services’ understanding of what victims of violence needed and the knowledge and expertise of women's shelters. It also indicates that the expanded capability of social services to procure placements for shelter contributes to contradictory and conflicting definitions of what shelter is and what a woman exposed to domestic violence needs (Prop 2006/07:38).

The Women's Shelter Movement's unclear role is framed as a problem that was resolved by policy solutions in favor of professionalization and increased regulation of shelters. This change altered the understanding of what a shelter was, from being a specific type of (nonprofit) organization to being a type of service. Consequently, “shelter” was separated from women's shelters, as they were becoming influenced by governmental regulations and state definitions rather than being shaped by the Women's Shelter Movement. Thus, the analysis reveals that the policy limits the operational scope of women's shelters and co-opts the mandate to define what constitutes a shelter.

The separation of the Women's Shelter Movement from the shelter as an integrated service became especially clear when the policy texts emphasized that, despite state regulations, women's shelters had the freedom to organize and shape other parts of their operations independently (e.g., free counseling services, violence prevention, and advocacy work). Through state policy, the government assumed ultimate responsibility for ensuring that victims of violence were offered shelter, in line with international conventions regarding the state's obligation to eliminate men's violence against women (Stubberud et al., 2018; Wemrell et al., 2020). However, the impact of institutionalization can also be understood as hampering the nonprofits, as they could only continue operating on the premises and requirements set by the state, regardless of whether the operation had been successfully conducted for 40 years or was newly established.

The public funding for shelters has also contributed to a redefinition and fragmentation of the Women's Shelter Movement. Women's shelters have historically relied on grants, and it was established early in policy that public resources should fund shelters, with particular emphasis on safeguarding the independent and nonprofit character of the Women's Shelter Movement. However, this led to ambiguities regarding the division of responsibility, as the grants were intended, among other things, to help expand the workforce and develop operations into more professionalized entities (Prop 1997/87:55). This created a seemingly contradictory problem representation arising in the mid-2000s; on the one hand, policy emphasized increased funding for women's shelters to strengthen their societal role and ensure protection and support for victims of violence (Prop 2005/06:155; SOU 2004:121), while on the other hand, questions arose regarding what value funders were receiving for their money, which reduced the autonomy of the Women's Shelter Movement (SOU 2006:65; SOU 2007:66).

The lack of transparency in women's shelters was depicted as a problem with the argument that no other welfare services would be purchased without some form of quality assurance (SOU 2006:65). Contrary to previous policy, this framing opened the door to conditioning funding for women's shelters. These new conditional funding measures further contributed to a shift toward professionalization and institutionalization, and today, since shelter is now a commodified service within a market, it can no longer be funded with grants. This development can also be understood as a response to the Istanbul Convention, where Sweden was encouraged to ensure grants for non-profit women's shelters to fulfill the convention (Stubberud et al., 2018). Stubberud et al. (2018) also showed a need for Sweden to strengthen its governance in the field to meet the demands of the convention effectively.

Conditional grants reinforce the conclusion that government policy has led to a redefinition of women's shelters, which can now be described as divided into two parts: one part consists of women's shelters that remain free, independent associations advocating for abused women and children. In contrast, the other part (shelter branch) is subordinate to state regulation and defined by government requirements and frameworks rather than the needs of abused women and children. Although this development is observed in several parts of the Nordic countries, concerns exist regarding this fragmentation, as the preventive work is closely interconnected with the services within the shelter, as well as its funding (Stubberud et al., 2018)

Lastly, the concept of quality has been a central part of the arguments for developing and reshaping shelter as an intervention. The state's current regulation of shelters was finalized in 2024 with the launch of a shelter reform, aimed at ensuring shelter quality. The reform required shelters to apply for licensing and meet specific quality criteria (Prop 2023/24:31). Previous research indicates that licensing rarely achieves the intended quality effect (Pålsson, 2018; Sallnäs & Wiklund, 2018). On the contrary, previous studies suggest that political measures such as licensing are instead a tool for managing the market and shaping the applicants (Davidson, 2009; Power, 1999). This can occur by the conditions for the license compelling applicants to design their shelter and interventions to match the state requirements (rather than the client's needs) through, for example, quality indicators and training requirements for staff (Pålsson, 2018), or by the state gate-keeping the power over who is allowed entry into the market (Power, 1999), without a guarantee that unscrupulous actors are filtered out (Pålsson, 2018).

What was not addressed was that the development of quality assurance and follow-up comes with reduced autonomy and increased auditing. With additional quality assurance requirements, the need arises for social services to audit and control procured services, as well as to regulate the requirements, criteria, and conditions governing shelter interventions. In this way, the state's problem representation that led to the establishment of quality assurance can be seen as a multi-layered governance measure; on one hand, the stated ambition was that the quality assurance would enhance the status of women's shelters and to ensure appropriate placements for victims of violence, and on the other, to implement further control and regulation of shelters.

A discernible transition transpires from the perception that the Women's Shelter Movement possesses both the knowledge and the mandate to devise interventions for victims of violence, to the gradual assumption of this responsibility by the state. Furthermore, it is noteworthy that the calls for quality assurance, which catalyzed this shift in power concerning mandates and interpretive authority, emerge from the deficiencies within social services (e.g., questionable accommodation in hotels and campsites) rather than as a consequence of actions taken by the Women's Shelter Movement. A more comprehensive analysis may even interpret the rhetoric present in the policy texts as a façade, wherein the state purports to enhance the Women's Shelter Movement under the guise of quality assurance, yet in practice, this results in the diminishment of the Women's Shelter Movement into an entity subject to oversight, a situation in which the demands and conditions imposed by the authorities overrule the movement's experiences and ideological framework. The solution to the problem representation of shelter quality requiring improvement has led to a system of shelters characterized by measurability, auditing, and evaluation.

## Conclusion

The analysis shows that the problem representation regarding women's shelters’ capacity to provide support originated from the division of responsibilities between the Women's Shelters Movement and social services. Early policy documents showed that social services were responsible for victims of domestic violence, which coincided with the Women's Shelter Movement's aim to integrate them into the welfare system (Eduards, 2002; Hague, 2021; Helmersson, 2017). Despite clarifying the municipality's responsibilities, the public sector did not expand its shelters for abused women. Instead, the municipalities continued to use women's shelters to ensure support and protection for victims of violence.

The social services’ reliance on women's shelters was not controversial; instead, women's shelters were recognized as both authorities in their domain and as a viable solution that corresponded with the increasingly prominent NPM paradigm, which promoted market solutions. However, for this to be a sustainable long-term solution, women's shelters were compelled to institutionalize and formalize, a trend apparent in the analyzed policy reports. This transition occurred gradually, yet the shift from the movement's early, ideologically driven structure to the current, legally regulated framework is evident in the analysis. Shelters are now defined by measurement, audits, and evaluations aimed at ensuring and improving the quality of shelters.

The representations of problems concerning the capabilities of volunteers, the lack of transparency, the necessity to ensure quality, and the ambiguous delineation of responsibilities collectively underpinned policy solutions that led to a state-controlled welfare market, ultimately relegating women's shelters to functioning as a service within this market.

For the Women's Shelter Movement, this development has resulted in a diminished role as experts in the field and as the primary providers of support and protection for victims of violence, relegating them to a peripheral position within social services. Consequently, state regulations concerning shelters have necessitated an internal subdivision of women's shelters, whereby the shelter services are governed by law. At the same time, other functions may persist independently under a feminist ideology. This suggests that women's shelters must be primarily designed and operated following the social services’ frameworks and mandated licensing requirements, prioritizing statutory obligations and quality standards over the immediate needs of abused women and children. It remains uncertain whether the Women's Shelter Movement can, or indeed wishes to, continue offering shelter under these new stipulations.

For abused women, this shift signifies that access to protection has become increasingly limited, as it is no longer feasible for a woman with children to approach a women's shelter directly. Thus, governmental policies have influenced and redefined both the role and operational scope of the Women's Shelter Movement and fundamentally altered the system available for abused women seeking refuge from violence in intimate relationships. However, the recent reforms are also commendable, as they seek to enhance protection and elevate the quality of shelter. The eventual achievement of this objective can only be ascertained over time.
